# Synthetic symbiosis between a cyanobacterium and a ciliate toward novel chloroplast-like endosymbiosis

**DOI:** 10.1038/s41598-023-33321-w

**Published:** 2023-04-13

**Authors:** Yuki Azuma, Saburo Tsuru, Masumi Habuchi, Risa Takami, Sotaro Takano, Kayo Yamamoto, Kazufumi Hosoda

**Affiliations:** 1 Institute for Transdisciplinary Graduate Degree Programs, Osaka University, 1-5 Yamadaoka, Suita, Osaka 565-0871 Japan; 2 Graduate School of Information Science and Technology, Osaka University, 1-5 Yamadaoka, Suita, Osaka 565-0871 Japan; 3 Division of Hygienic Chemistry, Osaka Institute of Public Health, 1-3-3 Nakamichi, Higashinari-ku, Osaka, 537-0025 Japan; 4 Universal Biology Institute, Graduate School of Science, The University of Tokyo, 7-3-1 Hongo, Bunkyo-Ku, Tokyo, 113-0033 Japan; 5 Graduate School of Frontier Biosciences, Osaka University, Suita, Osaka 565-0871 Japan; 6 RIKEN Center for Biosystems Dynamics Research, 6-2-3 Furuedai, Suita, Osaka 565-0874 Japan; 7 Center for Information and Neural Networks (CiNet), National Institute of Information and Communications Technology (NICT), Osaka, Japan

**Keywords:** Ecology, Evolution, Systems biology

## Abstract

Chloroplasts are thought to have co-evolved through endosymbiosis, after a cyanobacterial-like prokaryote was engulfed by a eukaryotic cell; however, it is impossible to observe the process toward chloroplasts. In this study, we constructed an experimental symbiosis model to observe the initial stage in the process from independent organisms to a chloroplast-like organelle. Our system of synthetic symbiosis is capable of long-term coculture of two model organisms: a cyanobacterium (*Synechocystis* sp. PCC6803) as a symbiont and a ciliate (*Tetrahymena thermophila*) as a host with endocytic ability. The experimental system was clearly defined, because we used a synthetic medium and the cultures were shaken to avoid spatial complexity. We determined the experimental conditions for sustainable coculture, by analyzing population dynamics using a mathematical model. We experimentally demonstrated that the coculture was sustainable for at least 100 generations, through serial transfers. Moreover, we found that cells isolated after the serial transfer improved the probability of coexistence of both species without extinction in re-coculture. The constructed system will be useful for understanding the initial stage of primary endosymbiosis from cyanobacteria to chloroplasts, i.e., the origin of algae and plants.

## Introduction

Chloroplasts are considered to have evolved from a cyanobacteria-like prokaryote that originally lived independently and was engulfed by a eukaryotic cell through endocytosis^1–3^. Both the original host and symbiont are supposed to have adapted to the intracellular-endosymbiotic state through various changes, such as endosymbiotic gene transfer and genome shrinkage^4,5^. An understanding of the evolutionary process, from cyanobacteria-like cells to chloroplasts, has been achieved largely based on the comparison of existing cyanobacteria, chloroplasts, and nuclei of plant cells. The evolutionary process in which two independent organisms become one would consist mostly of gradual changes; however, the process involves an important stage in which a free-living organism becomes able to live inside another organism, which would be considered a rather momentary event relative to its long evolutionary history. It is challenging to investigate the past momentary event with only information obtained from limited remaining species that currently exists.

To investigate the details of intermediate states in the process from an independent organism to an organelle, it is effective to observe naturally existing endosymbioses, e.g., those between a ciliate (*Paramecium bursaria*) and a green alga (*Chlorella variabilis*)^6^; a ciliate (*Tetrahymena utriculariae*) and a green alga (*Micractinium* sp.)^7^; and a flagellate (*Hatena arenicola*) and a green alga (*Nephroselmis* sp.)^8^. These endosymbioses in nature would have undergone interactions between two species through a long period of coexistence to reach their current relationship. These endosymbioses have been revealed in detail through morphological observations, genetic analyses, and biochemical analyses. It is also possible to remove symbionts from the host and reconstitute intracellular endosymbiosis by mixing them^9^. However, these endosymbioses do not enable us to observe the initial stages including the encounter of two independent species.

To observe the initial stage in the process from the encounter, combining synthetic symbioses and experimental evolutions is a powerful strategy^10–12^. In particular, in a three-species system that consists of a green alga (*Micractinium* sp.), bacterium (*Escherichia coli*), and ciliate (*Tetrahymena thermophila*), it is known that a portion of the algal cells in a flask became more endosymbiotic with the ciliate cells, while another part of the algal cells became more ectosymbiotic with the bacterium, through a 5-year evolution^13^. This synthetic symbiosis will provide further information on the initial stage, in addition to the information already obtained from the natural endosymbiosis of *T. utriculariae* and *Micractinium* sp.^7^. Thus, synthetic symbioses in which constituent species can coexist stably for a long time provide an opportunity to investigate the evolutionary enhancement of interspecies interactions in real time.

However, in most studies of natural and synthetic endosymbioses between a photosynthetic symbiont and an endocytotic protozoa, the symbionts are eukaryotic algae, and thus, these symbiogeneses correspond to secondary endosymbiosis. There have been other synthetic experimental systems that have involved eukaryotes and cyanobacteria^14–16^; however, these studies did not focus on endocytosis, but instead on extracellular symbioses or the artificial introduction of cyanobacteria into cells.

In this study, we aimed to create a system for synthetic symbiosis that is capable of long-term cultivation for experimental evolution, using a cyanobacterium (*Synechocystis* sp. PCC6803) as a symbiont and a ciliate (*T. thermophila*) as a host capable of endocytosis. Both species are well-known model organisms, with information on their cell preservation methods and genome available^17–19^, which are necessary conditions for experimental evolutions to compare evolved cells with original cells. A chemically defined medium for the purpose of culturing both species has been proposed^20,21^, which is important because chemically defined media enable us to analyze whole chemical interactions in the synthetic system. This proposed chemically defined medium was rather nutritious and toxic to cyanobacteria, but a modified cyanobacteria strain that is suitable for the medium has been obtained through monocultural experimental evolution^21^. This adaptation of a cyanobacterium to a nutritious environment would be one of the necessary steps toward endosymbiosis. However, long-term coculture between the modified cyanobacterium and ciliate is yet to be achieved.

In the present study, we identified conditions in which long-term coculture of *Synechocystis* sp. PCC6803 and *T. thermophila* can be maintained by analyzing the population dynamics of the coculture using a mathematical model. The conditions were strict, because there were several factors that caused the system to break down. We demonstrated a 100-generations subculture with the co-existence of both species, which indicated that the conditions we identified were valid. We also found that the cells of both the species after 100 generations grew more stably in coculture than their original cells. Based on our results, a system for synthetic symbiosis that is useful for understanding the initial stage of chloroplast evolution is now available, which makes it possible to continuously observe the process of primary endosymbiosis, *i.e.*, the origin of algae and plants.

## Results and discussion

### Growth characteristics of *Synechocystis* sp. PCC6803 and *T. thermophila* in monocultures

We designed a synthetic symbiosis system that is composed of a cyanobacterium (*Synechocystis* sp. PCC6803, an evolved strain created in the previous study^21^) and a ciliated protozoan (*T. thermophila*). All cultures were illuminated for cyanobacterial photosynthesis and mixed well to simplify population dynamics, by ignoring the spatial structure. We used the synthetic medium TCM1_Glc-_ (“Materials and methods” section, Table S1). This medium is obtained by removing glucose, a primary carbon source, from TCM1, where the cell populations of the cyanobacterium and the ciliate can grow independently. TCM1 was obtained by adding some components of BG11, which is the minimum medium for the cyanobacterium, to CDM15, which is the minimum medium for the ciliate, modified from a standard chemical defined medium^22^. Glucose was omitted from TCM1, to enhance the nutrient dependency of the ciliate on the cyanobacterium.

**Figure 1:**
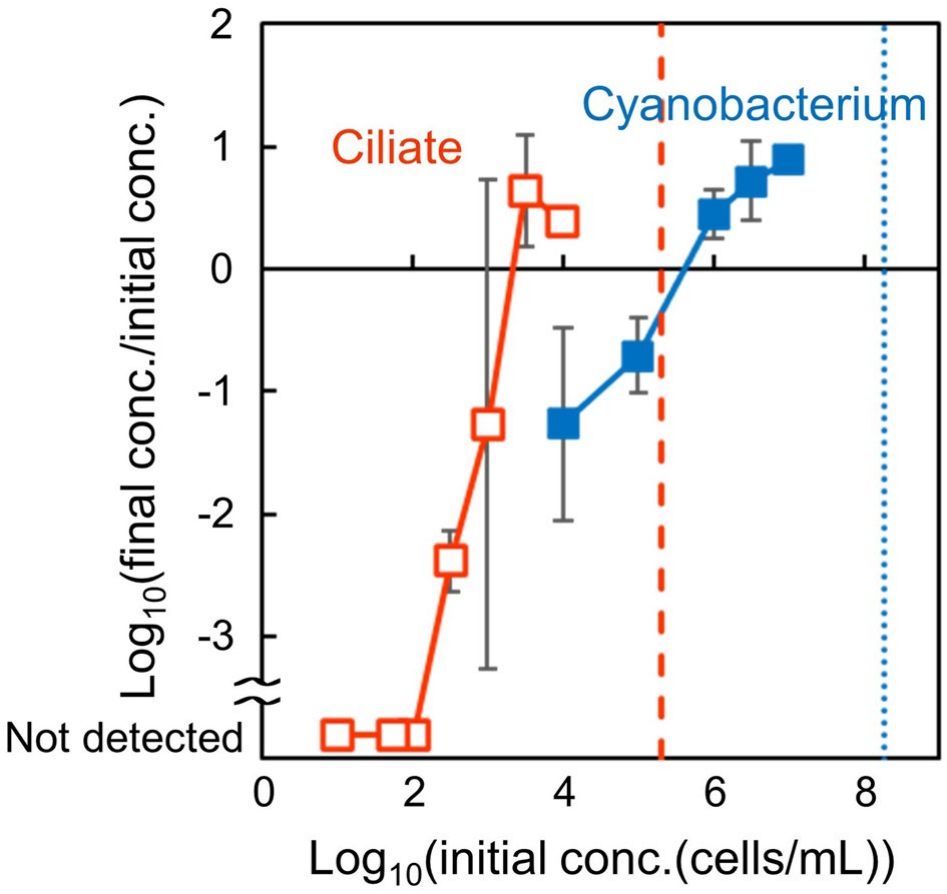
Growth characteristics of the cyanobacterium and ciliate cell populations in monocultures using TCM1_Glc−_. Blue-closed and red-open squares show the fold-changes in the cell concentration, after 3.5 days of culture of the cyanobacterium and ciliate, respectively. Each plotted data is the mean of at least 2 independent cultures, and the error bars indicate the standard deviation. Specifically, for the initial concentrations of the ciliate, 10^1^, 10^1.75^, 10^2^, 10^2.5^, 10^3^, 10^3.5^, and 10^4^ cells/mL, the number of experimental replicates were 2, 4, 2, 10, 4, 3, and 3, respectively. For the initial concentrations of the cyanobacterium, 10^4^, 10^5^, 10^6^, 10^6.5^, and 10^7^ cells/mL, the number of experimental replicates were 4, 4, 4, 15, and 3, respectively. The blue-dotted and red-dashed lines are the saturation concentrations of monocultures of the cyanobacterium in TCM1_Glc−_ and the ciliate in TCM1, respectively, and thus having an initial concentration higher than the tested concentration is not applicable for subculture experiments like experimental evolution.

We confirmed that the cyanobacterial cell population grew in monocultures using TCM1_Glc−_, when the initial cell concentration was higher than 10^6^ cells/mL (Fig. 1) (the population increase at 10^6^ cells/mL was significant; *t*-test, *DF* = 3, *α* = 0.05). This concentration-dependence has been revealed in a previous study^21^, and has been shown to be due to the medium, in which some components (such as amino acids) are still toxic to the cyanobacterium (but necessary for the ciliate). The growth of the ciliate cell population also showed concentration-dependence in monocultures using TCM1_Glc−_. The cell concentration decreased when the initial cell concentration was not higher than 10^2.5^ cells/mL. Such ciliate mortality at low concentrations has been reported previously^23^. The cell concentration increased slightly when the initial cell concentration was 10^4^ cells/mL (the increase was significant; *t*-test, *DF* = 3, *α* = 0.05), and we did not detect a significant increase or decrease in those intermediate ranges. The slight growth at 10^4^ cells/mL in the medium without glucose could be because the ciliate used other substances, such as citrate, as the carbon source. As mentioned above, the growth of the cell populations of both the species was concentration-dependent (higher was better, unless near saturation) in monocultures.

### Interactions in cocultures

**Figure 2:**
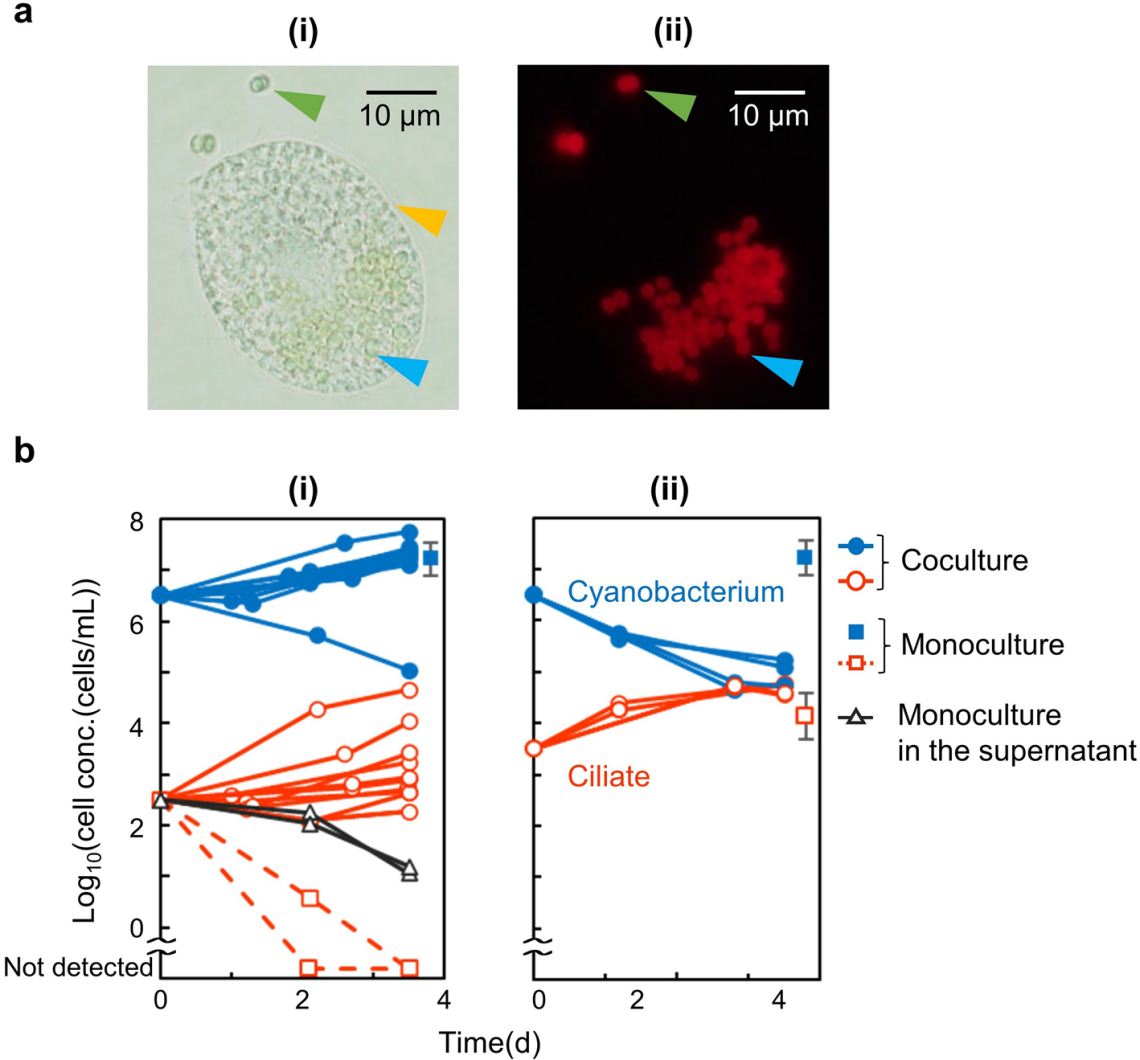
Interactions between the cyanobacterium and ciliate. (**a**) Representative micrographs of a ciliate cell that has taken up cyanobacterial cells. We sampled from a 26-h coculture, at initial concentrations of 10^4^ cells/mL ciliate and 10^8^ cells/mL cyanobacterium. Conditions of higher cell concentration were set, to easily observe the ciliates under the microscope. (**i**) A micrograph in bright field. (**ii**) A micrograph in fluorescence field. Red particles indicate autofluorescence derived from cyanobacterial chlorophyll. The orange arrow indicates the outline of the ciliate cell. The blue and green arrows indicate the cyanobacterial cells inside and outside the ciliate cell, respectively. (**b**) Cell population dynamics in cocultures. Blue-closed and red-open circles show the cell concentrations of the cyanobacterium and ciliate in the cocultures, respectively. Blue-closed and red-open squares indicate the cell concentrations of the cyanobacterium and the ciliate in monocultures, respectively. For the monoculture results, time courses are shown for the ciliate cell concentration in (**i**), to show the ciliate mortality at low concentration, while mean cell concentration at 3.5 d (the same results as in Fig. 1; for comparison) are shown (at 3.8 days in these graphs) for the other monoculture experiments, i.e., the ciliate in (**ii**) and the cyanobacterium in (**i,ii**). Black-open triangles indicate the cell concentration of the ciliate in monoculture, in which the supernatant of the monoculture of the cyanobacterium was used as the culture medium.

We investigated the effects of interactions between the two species. First, we found that the ciliate engulfed the cyanobacteria (Fig. 2a), as confirmed by the observation of cyanobacterial cells inside the ciliate cells.

Next, we investigated the effects of these interactions on cell population dynamics. When the initial ciliate cell concentration was 10^2.5^ cells/mL (Fig. 2b-(i)), no significant difference was observed in the cyanobacterial cell concentrations between the cocultures (blue-closed circles at 3.5 days) and monocultures (blue-closed squares; *t*-test, *DF* = 25, *α* = 0.05). On the other hand, when the initial ciliate cell concentration was 10^3.5^ cells/mL (Fig. 2b-(ii)), the cyanobacterial cell concentration in the cocultures (blue-closed circles at 3.5 days) was significantly lower than that in the monocultures (blue-closed square; *t*-test, *DF* = 17, *α* = 0.05), which suggests the effect of predation of the cyanobacterial cells by the ciliate cells. At the same time, the growth of the ciliate cell population was significantly greater in the cocultures (red-open circles with lines in Fig. 2b) than in the monocultures (red-open squares with dashed lines in Fig. 2b-(i) and without lines in Fig. 2b-(ii); one-tailed *t*-test, *DF* was 5 and 20, at initial concentrations of 10^3.5^ cells/mL and 10^2.5^ cells/mL, respectively, *α* = 0.05). These results suggested that ciliates utilized cyanobacteria for their growth, through predation.

When the initial cell concentration of the ciliate was 10^2.5^ cells/mL, the decrease in the population of ciliates that was observed in the monocultures (red-open squares with dashed lines in Fig. 2b-(i)) was found to be prevented in the cocultures (red-open circles with lines in Fig. 2b-(i)). These results suggested that the cause of ciliate mortality at low cell concentrations that was observed in the monoculture was eliminated by cyanobacteria. Indeed, we confirmed that the supernatant of the cyanobacterium monoculture reduced ciliate mortality at low cell concentrations (black-open triangles with lines in Fig. 2b-(i)).

As mentioned above, we found the population decrease of the cyanobacterial cells and the population growth of the ciliate cells, which would be due to predation and the avoidance of ciliate mortality by the cyanobacterium. All these interactions were dependent on the cell concentrations in the cocultures as well as monocultures. The population growth of both the species was better at a higher cell concentration in the monoculture, but a higher cell concentration of the ciliate led to a decrease in the cyanobacterial cell concentration in the coculture. Therefore, it is necessary to quantitatively understand the cell population dynamics of the coculture, to determine the conditions for long-term co-existence.

### Investigation of coculture conditions using a mathematical model of cell population dynamics

To find out the experimental conditions for the long-term co-existence of both species, i.e., symbiosis, we determined the range of initial cell concentrations of the cocultures, where the two species can be sustained throughout serially transferred cocultures. As mentioned above, changes in the cell populations of both the species depend on cell concentration. To quantitatively understand the cell population dynamics, we formulated a simple mathematical model for population dynamics, using a standard Monod function and concentration-dependent mortalities to match the experimental observations described above:

1 $$\begin{array}{l}\frac{\mathrm{d}{C}_{S}}{\mathrm{dt}}={k}_{S}{C}_{S}-{d}_{S}\frac{{C}_{S}{C}_{T}}{{C}_{S}+{K}_{M}}\\ \frac{\mathrm{d}{C}_{T}}{\mathrm{dt}}={k}_{T}\frac{{C}_{S}{C}_{T}}{{C}_{S}+{K}_{M}}-{d}_{T}\frac{{C}_{T}}{{{K}_{I}C}_{S}+{C}_{T}}\end{array},$$

where *C*, *k*, *d* are the cell concentration, rate constant of population growth, and rate constant of mortality, respectively, of the cyanobacterium *Synechocystis* sp. PCC6803 (subscript S) and the ciliate *T. thermophila* (subscript T). *K*_*M*_ is the Monod constant of the predation and *K*_*I*_ is the inhibition constant of ciliate mortality by the cyanobacterium.

For cyanobacterial cell population changes, we considered the effects of ciliate predation, in addition to independent growth. For ciliate cell population changes, we considered predation-dependent growth, self-concentration-dependent mortality, and inhibition of the ciliate mortality by cyanobacteria. All of these terms were assumed based on the experimental results shown in Fig. 2. This model is a simplified version, which includes only the terms necessary to investigate the target conditions of the coculture and ignores other effects such as the concentration-dependent growth of the cyanobacterium and cyanobacterium-independent slight growth of the ciliate. We confirmed that a more detailed model including these ignored terms could explain all the experiments carried out well, and both models gave similar results around the target conditions (Supplementary Fig. S1).

**Figure 3:**
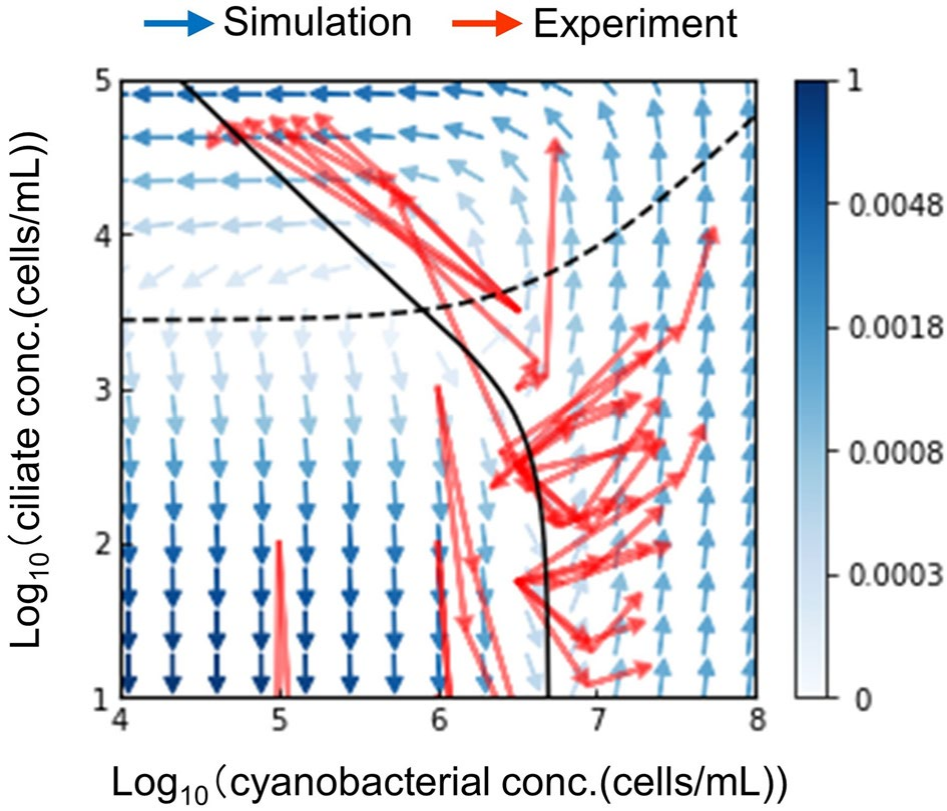
Direction field of the population dynamics of coculture. The red and blue arrows represent the values obtained from the experimental results and mathematical model, respectively. In each red arrow, the starting point indicates the initial cell concentration of both species in a coculture, while the ending point of the arrow indicates the cell concentration after 3.5 d. In each blue arrow, the angle shows the direction of change in the log-scale, as the vector of (*dC*_*S*_/*dt*)/*C*_*S*_ and (*dC*_*T*_/*dt*)/*C*_*T*_ from Eq. (1). The color of the blue arrows shows the magnitude of the vector in logarithm. The values of the model constants were obtained by fitting of the model to the experimental results, using the quasi-Newton method: specifically, the values of *k*_*S*_, *d*_*S*_, *k*_*T*_, *d*_*T*_, *K*_*M*_, and *K*_*I*_ were 0.285 d^–1^, 5.16 × 10^2^ d^–1^, 3.20 d^–1^, 1.50 × 10^3^ d^–1^, 4.99 × 10^6^ cells/mL, and 1.86 × 10^–4^, respectively. The black solid and dashed lines represent nullclines of population of the ciliate (*C*_*T*_ = *d*_*T*_(*C*_*S*_ + *K*_*M*_)/*k*_*T*_*C*_*S*_ $-$ *K*_*I*_*C*_*S*_) and cyanobacterium (*C*_*T*_ = *k*_*S*_(*C*_*S*_ + *K*_*M*_)/*d*_*S*_), respectively. For example, the black solid line denotes the boundary between increase or decrease of the ciliate population; the ciliate population increases (decreases) when the state is to the right (left) of the line. Similarly, the black dashed line denotes the boundary between increase or decrease of the cyanobacterial population; the cyanobacterial population increases (decreases) when the state is below (above) of the line.

We tested the cocultures with various initial concentrations near the predicted boundary, where both cell populations increased. The values of the model constants were determined by fitting the model to the experimental results using the quasi-Newton method. Figure 3 shows the direction field calculated using the mathematical model, with the fitted constants (blue arrows) overlaid on the experimental results (red arrows). This model explains the experimental results well.

The model shows that there is no stable equilibrium point or orbit in this primitive symbiotic system, where the two species co-exist unless the system is externally controlled, such as by serial transfers. Therefore, it is necessary to find the condition in which both the species increase during serial transfers. We found that the cell populations of both the species increased after 3.5 days in cocultures, when the initial cell concentrations of the cyanobacterium and ciliate were 10^6.5^–10^7^ cells/mL and 10^2^–10^3^ cells/mL, respectively.

Below, we explain the interpretation of these experimental results using this model. The cyanobacterial population increases when predation by ciliates is not too high (Fig. 3, when the ciliate concentration is lower than the black-dashed line). The ciliate population increases when there is a sufficient concentration of the cyanobacterium for predation (Fig. 3, when the cyanobacterial concentration is higher than the black-solid line). Therefore, a balanced amount of predation is required, to allow for an increase in both the populations. When the initial cell population falls outside the range of the conditions obtained above, at least one population fails to increase, as follows: when the cyanobacterial concentration was higher than 10^7^ cells/mL, ciliates grew rapidly, and their predation reduced the cyanobacterium steeply. When the cyanobacterial concentration is lower than 10^6.5^ cells/mL, the ciliate population decreases, because predation is insufficient. When the ciliate concentration is higher than 10^3^ cells/mL, the cyanobacterial population decreases because of excessive predation. When the ciliate concentration is lower than 10^2^ cells/mL, there is no problem in this model of population dynamics, but extremely low concentrations are generally problematic in the practical scenario, because they are undetectable, and discreteness makes the experiments unstable. As described above, we obtained a range of possible conditions for the serially transferred cocultures and their interpretation from experiments and mathematical modeling.

### Serial transfers of the coculture

We demonstrated serial transfer of the coculture using the following procedure based on the above results. First, we started cocultures in which the initial cell concentration of the cyanobacterium was 10^6.5^ cells/mL and that of the ciliate ranged from 10^1.75^ to 10^3.5^ cells/mL. Of these cocultures, we transferred the cultures where the ciliate population increased and the cyanobacterial cell concentration was higher than 10^6^ cells/mL, after 3.5 days. The transfer was carried out by diluting the cocultures so that the cell concentration of the ciliate became the initial concentration of the next coculture. We did not control the initial cyanobacterial concentration at dilution. We set the initial ciliate cell concentration to have a range, because these experimental conditions are in the boundary region and unstable (as shown in Fig. 3). Because of the instability of the coculture, we increased the number of cultures at the time of transfer, so there were multiple lines in parallel.

**Figure 4:**
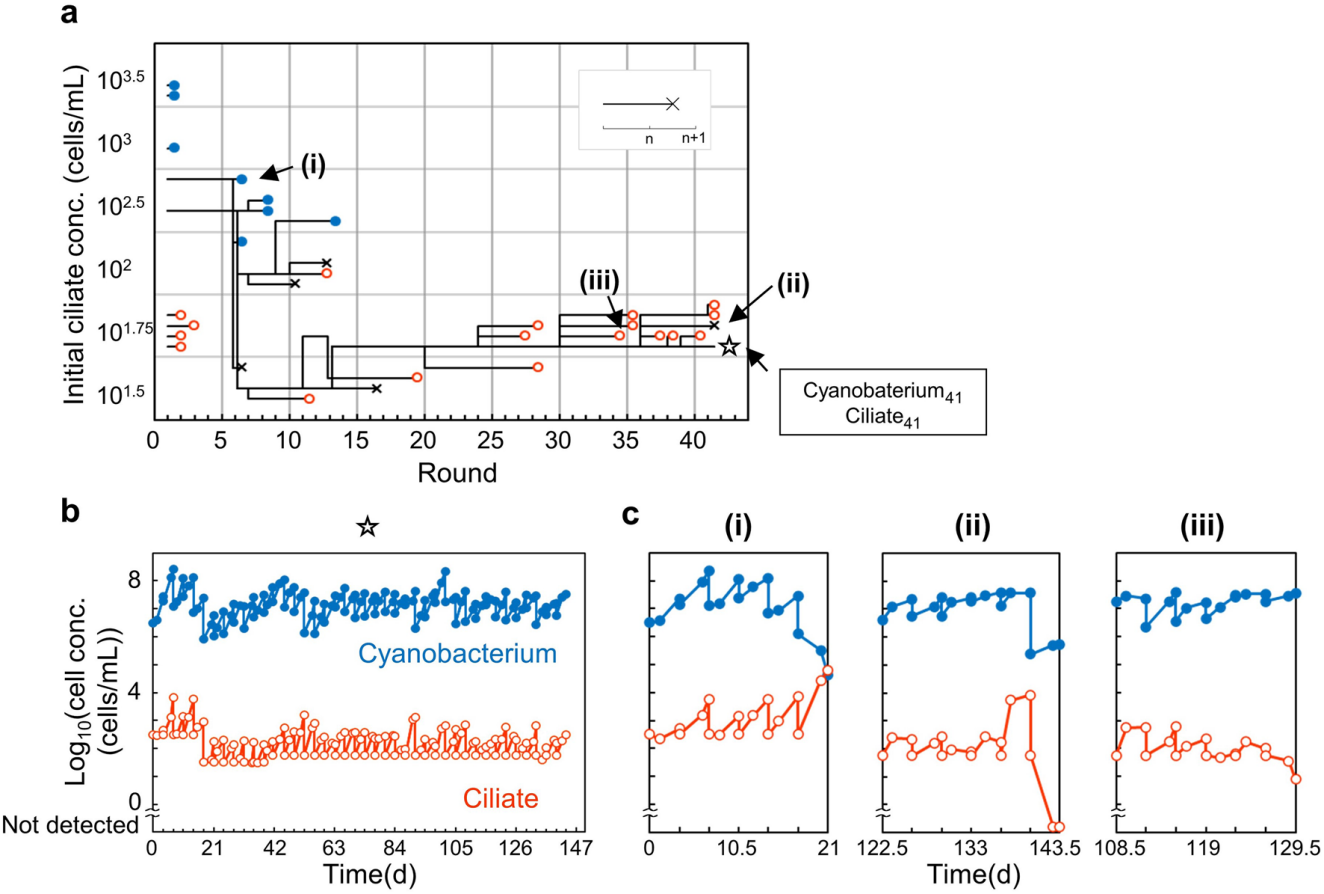
Serially transferred cocultures of the cyanobacterium and ciliate. (**a**) Family trees of the serially transferred cocultures showing the relation between parental cultures and their derivative lineages. Length of the black horizontal lines represents the duration from the start of the lineages to the end. The vertical axis is the initial cell concentration of the ciliate, but the values are not continuous. The initial concentrations within each ticks frame are the same, and the values are depicted (e.g., the initial concentration of the four lineages with red-open circles from round 1 are all 10^1.75^ cells/mL). The longest transfer (round 41) was denoted using a star, and the cells obtained from the end of this 41st-round coculture were designated as Cyanobacterium_41_ and Ciliate_41_. The causes of the stop of the propagation were distinguished using three symbols, blue-closed circles, black crosses, and red-open circles, corresponding to the three patterns shown in (**c**) (i), (ii), and (iii), respectively, as described in detail in the main text. A symbol at round *n* indicates that its lineage experienced round *n* and stopped before round *n* + 1. (**b**) Growth curves of the longest lineages. (**c**) Growth curves of representative lineages whose propagation had been stopped by different causes. The end-points of each growth curve are indicated using arrows in (**a**). Blue-closed and red-open circles correspond to the growth curves for the cyanobacterium and ciliate, respectively. (i) Cyanobacterial concentration decreased due to the ciliate overgrowth. (ii) Initial cyanobacterial concentration became too low due to high-magnification dilution. (iii) The ciliate population did not grow.

Figure 4a shows the history of serially transferred cocultures. We confirmed that the coculture shown in Fig. 4b was sustainable for at least 101 generations (143.5 days, 41 transfers; another independent line for reproducibility is shown in Supplementary Fig. S2). We categorized the patterns of transfer failure into three categories (Fig. 4c): (i) the ciliate concentration became too high and the cyanobacterial population decreased, (ii) a high-magnification dilution due to a large increase in the ciliate population made the next initial cyanobacterial concentration too low, and (iii) the ciliate concentration decreased. Pattern (i) was predicted using the model. Pattern (i) was observed mainly in the coculture with a high ciliate concentration (Fig. 4a) and was avoided when the initial ciliate concentration was lowered at transfer (round 6). Patterns (ii) and (iii) would be caused by instability, because these conditions are boundary conditions in the population dynamics. The coculture would become (ii) and (iii) when the growth of the ciliate was too large and small, respectively. Such variability might be due to the cell status of one or both species, as a hidden variable not included in the mathematical models. Although the culture was not stable, we established a method of long-term subculturing, by maintaining multiple lines with a range of initial ciliate concentrations.

### Re-coculture using isolated cells, after 101 generations of coculture

**Figure 5:**
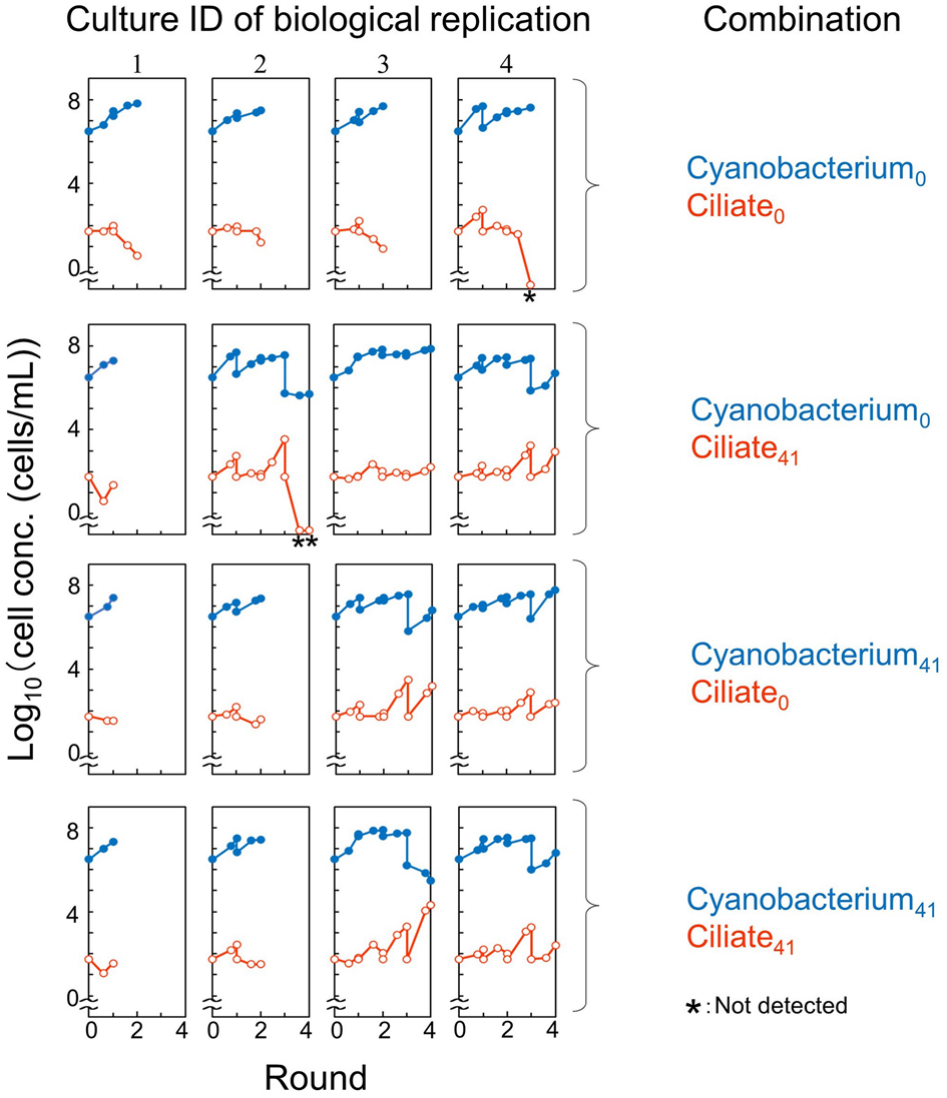
Improved sustainability through the serially transferred coculture. Growth curves of the cocultures of four possible combinations of the original (subscript 0) and evolved (subscript 41; isolated from round 41) cells of the cyanobacterium and ciliate (4 biological replicates are shown). The blue-closed and red-open circles correspond to the growth curves for the cyanobacterium and ciliate, respectively. The asterisks indicate that the ciliate was not detected.

The cells might have changed their growth characteristics in the 101 generations of coculture. We isolated and stocked post-transfer cyanobacterial and ciliate cells from the cocultures, after 41 transfers (referred to hereafter as evolved cells). We mixed these evolved cells with the original cells as re-cocultures. We set the initial ciliate concentration at 10^1.75^ cells/mL, which made it difficult for the pair of the original cells to maintain both cell populations. We found that both cell populations were maintained in some of the four tested replicates of the re-cocultures, in which at least one population, either the cyanobacterium or the ciliate, was the evolved cell (Fig. 5). The total maintenance proportions at round 3 of these evolved cells-included re-cocultures (7/12) was significantly different from that of the original cocultures (0/4; in these experiments, note that both populations were not maintained for at least an additional of 4 more original cocultures, as shown below), as assessed using the two-proportions *z*-test (*α* = 0.05). It was still a small change that was difficult to investigate further, but we found that some significant changes associated with coculture growth occurred within the 101 generations. At this time, it is not possible to determine, for example, whether new mutations were introduced during the 100 generations or adaptive individuals pre-contained within the initial population were selected. Further continuation of cocultures and analysis of genomic changes are expected in the future.

Evolutionary changes can be interpreted by using mathematical models. For example, as in this case, stable growth at low ciliate concentrations depending on the cyanobacterium may have increased the *K*_*I*_ in the model. From another point of view, in the nullcline (black solid line) of the ciliate in Fig. 3, the position when the ciliate concentration was low moved to the left. We can interpret this as an increase in the avoidance of ciliate mortality at low concentrations (black-open triangles with lines in Fig. 2b-(i) observed in monoculture). Thus, interpretation using the model contributes to verifying this mechanism. However, as mentioned above, this result is too small to verify. In addition, there is no stable equilibrium point in the novel symbiotic system constructed at present; however, an equilibrium point may appear with evolution. For example, considering that the concentration-dependence of the cyanobacterium in monoculture and the death of the ciliate at low concentrations are eliminated due to evolution, in addition to which the growth of the ciliate is saturated while assuming that the Hill coefficient is 2, as in the detailed model, there will be a condition where the equilibrium point appears at the center of the stable orbit in the mathematical model (Supplementary Figs. S1, S3). As mentioned above, our mathematical model is useful not only for system construction, as in this case, but also for understanding future evolution.

## Conclusions

We constructed a sustainable synthetic symbiosis system using a cyanobacterium (*Synechocystis* sp. PCC6803) and ciliate (*Tetrahymena thermophila*), which are not symbiotic in nature. The culture conditions required for long-term coculture were predicted using mathematical models and experimentally confirmed. We found that the coculture was sustainable for at least 101 generations (143.5 days, 41 transfers). Moreover, the cells isolated from the cocultures after 101 generations increased the sustainability of the coculture, suggesting that further coculture will lead to further development of the two species. Our synthetic symbiosis is well mixed, to ignore spatial structures, and is composed of known elements, such as model organisms and synthetic defined media. Thus, the experiment is clear, and we can track future evolutionary changes at various hierarchical levels, such as phenotypes, genotypes, and molecules. Therefore, our synthetic symbiosis would be one of the most convenient experimental models for simulating the initial stage in the process of primary endosymbiosis from cyanobacteria to chloroplasts, i.e., the origin of algae and plants.

## Materials and methods

### Strains and culture conditions

We used *Tetrahymena thermophila* cells, which were derived by mating CU427 and CU428. The parental strains were obtained from the National Tetrahymena Stock Center at Cornell University (Ithaca, NY, USA). After mating CU427 and CU428, the cells were cultured in Neff medium with 0.025 g/L of cycloheximide and 0.015 g/L of 6-methylpurine^19^. The cells were subcultured three times (100-fold growth each) and then freeze-stocked. The frozen stock was once inoculated into Neff medium. The recovered cells were washed and transferred into TCM1 and then used as described below. A derivative strain of *Synechocystis* sp. PCC6803 was used, which was previously obtained as an evolved strain of experimental evolution that is capable of adaptation to toxic amino acids in TCM1^21^ (Supplementary Table S1). The genome of this strain was analyzed previously^21^. The frozen stock was inoculated into TCM1_Glc−_ as described below. TCM1 is a synthetic medium based on CDM15 medium^20^ (for *T. thermophila*) and BG-11 medium^24^ (for *Synechocystis* sp. PCC6803). A derivative medium, TCM1 without glucose, denoted as TCM1_Glc−_, was used for the coculture of ciliates and cyanobacteria. For cell growth in ciliate monocultures, we used TCM1. Ciliate cells in these monocultures were washed twice with TCM1_Glc-_, by means of centrifugation at 600×*g* for 2 min, and then once transferred to another monoculture with fresh TCM1_Glc−_, for 3.5 days, to reset the ciliate cell status (Supplementary Fig. S4). The ciliate cells in monocultures using TCM1_Glc−_ were again washed by means of centrifugation with fresh TCM1_Glc−_, following which the washed cells were inoculated into the cocultures in TCM1_Glc−_. For cell growth in cyanobacterial monocultures, we used TCM1_Glc−_. Cyanobacterial cells in these monocultures were washed twice with TCM1_Glc−_, by means of centrifugation at 7000×*g* for 5 min, following which the washed cells were inoculated into the cocultures in TCM1_Glc−_. We used 6-well polystyrene microplates for coculture, with a culture volume of 3 mL, under continuous illumination (18 µmol/m^2^/s), at 30 °C, with orbital shaking (100 rpm).

### Measurement of cell concentrations

Cell concentrations at a high range (≥ 10^3^ cells/mL) of the ciliate were determined using a Fuchs–Rosenthal hemocytometer, after fixation, as described previously^20^. Cell concentrations at a low range (< 10^3^ cells/mL) of the ciliate were determined using a tube chamber (Supplementary Fig. S5). In brief, we fixed the cells and waited for them to sink to the bottom of the chamber. The number of cells that sunk was counted and divided by the applied volume of the sample, to calculate the cell concentrations. Cell concentrations of the cyanobacterium were determined using a flow cytometer (FACSCanto™ II; BD Biosciences, Franklin Lakes, New Jersey, USA) with a 488-nm argon laser. The sampled cell cultures were loaded after mixing with a known concentration of fluorescent beads (Fluoresbrite YG Microspheres, 6 µm; Polysciences Inc., Warrington, PA, USA), as detailed previously^21^. Long-pass (670 LP) and band-pass (515–535 nm) filters were used to detect the cyanobacterial cells and fluorescent beads, respectively.

### Serially transferred cocultures

Serially transferred cocultures were started, in a procedure similar to that of the normal coculture described above. The initial cell concentration of the cyanobacterium was fixed (10^6.5^ cells/mL), whereas that of the ciliate (denoted as *C*_T0_) varied from 10^1.5^ to 10^3.5^ cells/mL, as shown in Fig. 4a. We measured cell concentrations of the cocultures after 3.5 d (end of the first round) and transferred the cultures to fresh medium, by means of dilution, if the cell concentrations of the cyanobacterium (denoted as *C*_S_) and ciliate (denoted as *C*_T_) satisfied the defined criteria (*C*_T_ ≥ *C*_T0_ and *C*_S_ ≥ 10^6^ cells/mL). The former condition was required to initialize *C*_T_ at the next coculture, by means of dilution. The latter condition was required for stable cyanobacterial growth. This procedure was repeated for each transfer. We also generated multiple derivative lineages from the parental lineage, to maintain the serial transfer stably.

## Supplementary Information

Supplementary Information.

## Data Availability

All data generated or analyzed during this study are included in this published article (and its Supplementary Information files).
