# Supplementary material for: Synthetic symbiosis between a cyanobacterium and a ciliate toward novel chloroplast-like endosymbiosis
Source: Sci Rep. 2023 Apr 13;13:6104. doi: 10.1038/s41598-023-33321-w (PMC10102011; doi:10.1038/s41598-023-33321-w)
Supplement: Supplementary file 1 — Supplementary Information. [file 41598_2023_33321_MOESM1_ESM.pdf]

Supplementary information

Synthetic symbiosis between a cyanobacterium and a ciliate toward novel chloroplast-like endosymbiosis

Yuki Azuma, Saburo Tsuru, Masumi Habuchi, Risa Takami, Sotaro Takano, Kayo Yamamoto, Kazufumi Hosoda

This file includes

- Figures S1, S2, S3, S4, and S5
- Table S1

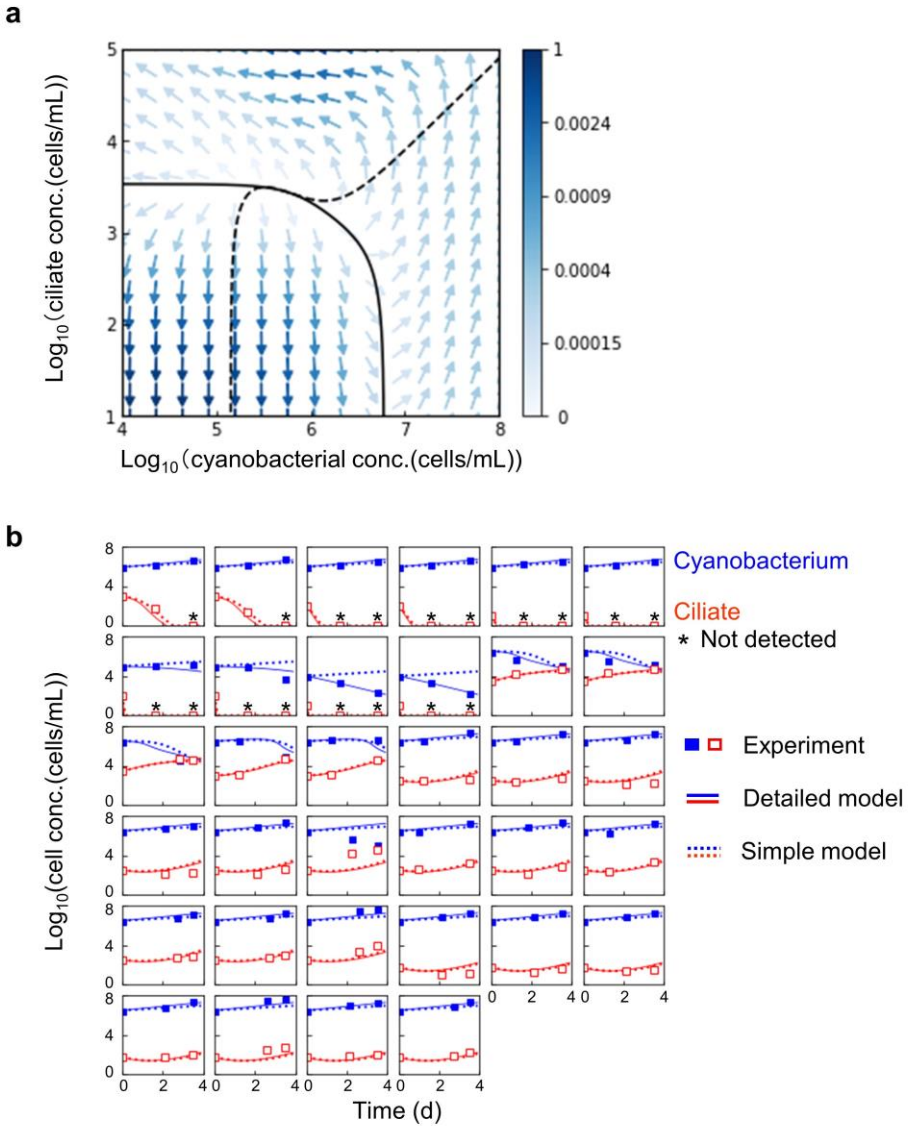

Figure S1. Detailed mathematical model. We built a mathematical model that better fits the actual data, by using Hill coefficient (h=2) and including two effects that were observed in the monocultures, but ignored in Eq. (1): (i) concentration-dependent cyanobacterial population growth (first term of the upper equation), and (ii) autonomous population growth of the ciliate (first term of the lower equation):

$$\frac{\mathrm{dC}_S}{\mathrm{dt}} = \left(\frac{k_S C_S}{C_S + K_{M1}} - d_{S1}\right)\mathrm{C}_S - d_{S2}\frac{C_S^h \mathrm{C}_T}{C_S^h + K_{M2}^h}$$
$$\frac{\mathrm{dC}_T}{\mathrm{dt}} = k_{T1}\mathrm{C}_T + k_{T2}\frac{C_S^h \mathrm{C}_T}{C_S^h + K_{M2}^h} - d_T\frac{\mathrm{C}_T}{K_I \mathrm{C}_S + \mathrm{C}_T} \qquad (2)$$

where C, k, d are the cell concentration, rate constant of population growth, and rate constant of

mortality, respectively, of the cyanobacterium *Synechocystis* sp. PCC6803 (subscript S) and the ciliate *T. thermophila* (subscript T). K_{M1} and K_{M2} are the Monod constants of autonomous cyanobacterial growth and predation of the cyanobacterium by ciliates, respectively. K_I is the inhibition constant of ciliate mortality by cyanobacteria. (**a**) Direction field of population dynamics derived from the mathematical model in Eq. (2). The meaning of the arrows and color intensity are the same as those in Fig. 3. The values of k_S, k_{T1}, k_{T2}, d_{S1}, d_{S2}, d_T, K_{M1}, K_{M2}, and K_I were 1.66 d^{-1}, 0.673 d^{-1}, 1.05 d^{-1}, 1.16 d^{-1}, 6.29×10^2 d^{-1}, 2.27×10^3 d^{-1}, 6.07×10^4 cells/mL, 1.61×10^6 cells/mL, and 2.30×10^{-4}, respectively. The black solid and dashed lines represent nullclines of the population in the ciliate, $C_T = [d_T(C_S^2 + K_{M2}^2) - K_I C_S\{k_{T2}C_S^2 + k_{T1}(C_S^2 + K_{M2}^2)\}] / \{k_{T2}C_S^2 + k_{T1}(C_S^2 + K_{M2}^2)\}$ and the cyanobacterium, $C_T = [(C_S^2 + K_{M2}^2)\ \{k_S C_S - d_{S1}(C_S + K_{M1})\} / d_{S2}C_S(C_S + K_{M1})]$, respectively. (**b**) Growth curves obtained from experiments and mathematical model. The blue and red squares correspond to the cell concentrations of the cyanobacterium and ciliate, respectively, obtained from the experiments. Asterisks indicate that no ciliates were detected. The solid and dotted lines are derived from Eqs. (2) and (1), respectively: The model in Eq. (2) fits the experimental data better than the model in Eq. (1); however, the obtained conditions for sustainable coculture were similar in these models. Therefore, we have chosen a simpler model for the main text.

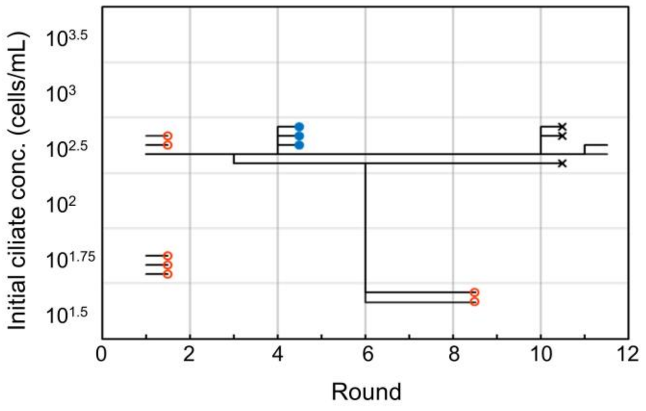

Figure S2. Another series of independent serial transfers of the coculture. Family trees of the serial transfer are shown, in a manner similar to that in Fig. 4a. The transfers of the cocultures were sustainable in this different series from Fig. 4a.

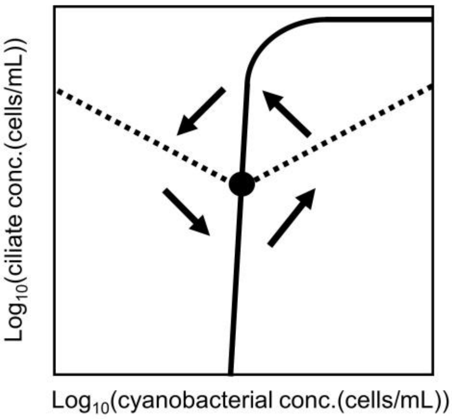

Figure S3. Schematic diagram of the direction field of the population dynamics under the condition that the equilibrium point appears. The closed point shows the equilibrium point discussed in the paragraph "Re-coculture using isolated cells after 101 generations of coculture". In the lower right region, if the orbit converges to a right dashed nullcline, a stable orbit appears around the equilibrium point. In each arrow, the angle shows the direction of the change in log-scale, as the vector of $(\mathrm{d}C_S/\mathrm{d}t)/C_S$ and $(\mathrm{d}C_T/\mathrm{d}t)/C_T$. The solid and dashed lines represent nullclines of the ciliate and cyanobacterium populations, respectively.

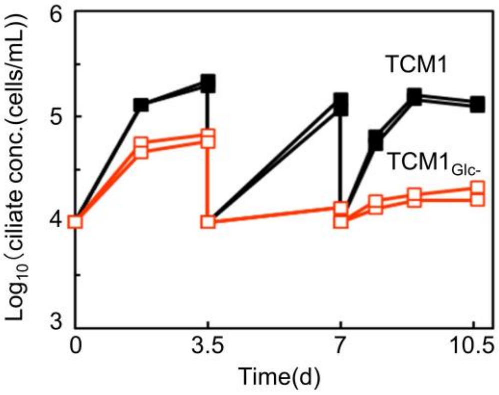

Figure S4. Carryover of glucose in the ciliate. Black-filled and red-open squares represent the growth curves of the ciliate cells in TCM1 and $TCM1_{Glc-}$, respectively. We pre-cultured the ciliate cells with TCM1 before these experiments. The ciliate cell concentration increased 6-fold after 3.5 d, due to carryover of glucose in the ciliate, even though we washed the medium twice using $TCM1_{Glc-}$ (see Methods for details). After 1 transfer in $TCM1_{Glc-}$, the ciliate increased slightly, because the ciliate used other substances in the medium as a carbon source.

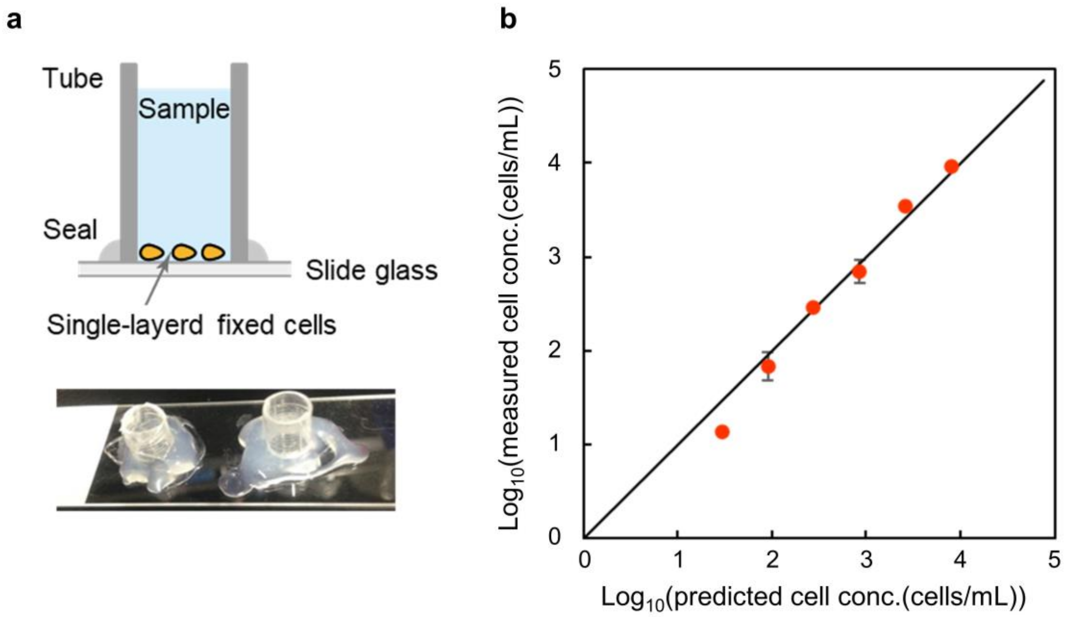

Figure S5. Measurement of the ciliate cells by using tube chambers. (**a**) The schematic diagram and the actual product. The chamber was made by cutting a tube and attaching it to a glass slide. The immobilized sample was placed in this tube, allowed to settle, following which the number of cells was counted under a microscope. (**b**) Correlation between predicted (from dilution) and measured cell concentrations. At least two independent dilution series, from $10^{1.5}$ to 10^{4} cells/mL, were prepared and measured. The black line is the ideal line.

Table S1. Components of $TCM1_{Glc-}$. TCM1 was prepared by adding glucose to $TCM1_{Glc-}$, at a final concentration of 27.8 mM.

Component	Concentration (mM)
L-Arg	1.42
L-His	0.954
L-Ile	1.52
L-Leu	1.52
L-Lys	1.09
L-Met	1.01
L-Phe	0.908
L-Ser	1.43
L-Thr	1.68
L-Trp	0.734
L-Val	0.854
Guanosine	7.1×10^{-2}
Uridine	8.2×10^{-2}
K_2HPO_4	1.10
KH_2PO_4	1.84
Tripotassium citrate	2.00
$MgSO_4 \cdot 7H_2O$	2.03
$CaCl_2$	6.8×10^{-2}
HEPES	20.0
EDTA・2Na	3.0×10^{-3}
H_3BO_3	4.6×10^{-2}
$NaNO_3$	17.6
Na riboflavin phosphate・$2H_2O$	9.7×10^{-4}
DL-6,8-thioctic acid	4.8×10^{-4}
Thiamine-HCl	1.5×10^{-3}
Pyridoxal-HCl	4.9×10^{-4}
Nicotinic acid	7.3×10^{-3}
D-Pantothenic acid, Ca-salt	3.4×10^{-3}
Folinic acid, Ca-salt	2.0×10^{-4}
$FeCl_3 \cdot 6H_2O$	3.7×10^{-2}
$MnSO_4 \cdot 4H_2O$	7.2×10^{-3}

$Co(NO_3)_2 \cdot 6H_2O$	1.7×10^{-3}
$ZnSO_4 \cdot 7H_2O$	1.6×10^{-2}
$CuSO_4 \cdot 5H_2O$	1.2×10^{-3}
$(NH_4)_6Mo_7O_{24} \cdot 4H_2O$	8.1×10^{-5}
Hemin	1.0×10^{-2}
